# Supplementary material for: Acute Toxicity of Metal Oxide Nanoparticles—Role of Intracellular Localization In Vitro in Lung Epithelial Cells
Source: Int J Mol Sci. 2025 Aug 30;26(17):8451. doi: 10.3390/ijms26178451 (PMC12428783; doi:10.3390/ijms26178451)
Supplement: Supplementary file 1 [file ijms-26-08451-s001.zip › ijms-3794761-supplementary.pdf]

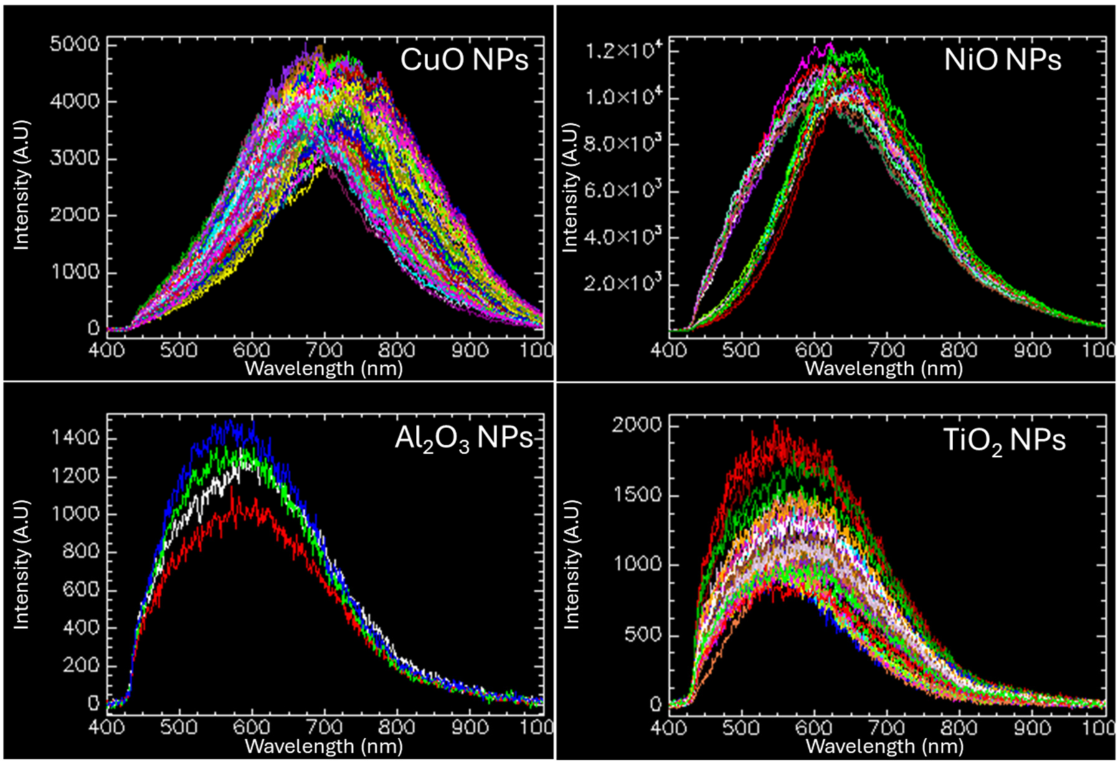

Figure S1. Filtered hyperspectral libraries of CuO, NiO, Al_2O_3, and TiO_2 NPs used in this study. Each library has 132, 22, 4, and 52 final spectra. Intensity is in arbitrary units.

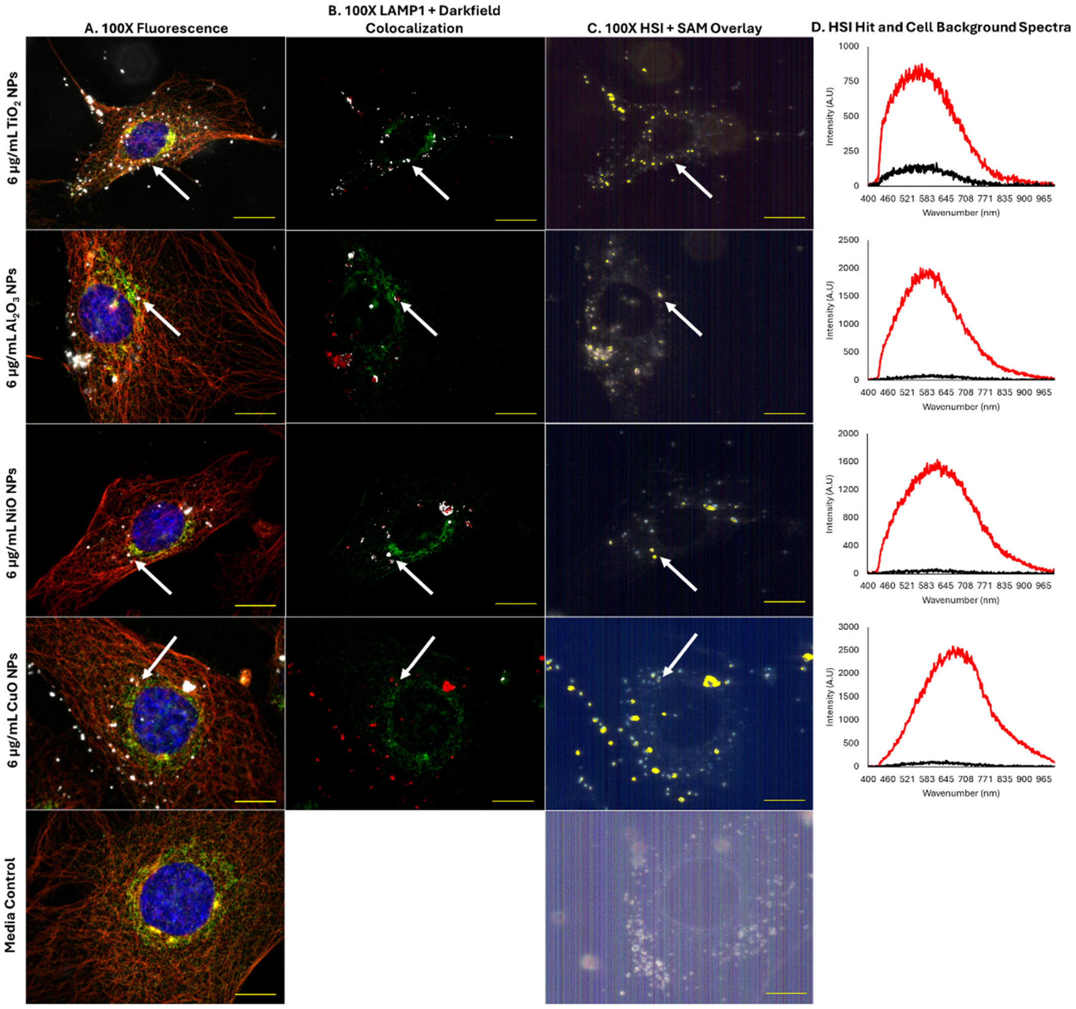

Figure S2. 100X enhanced darkfield fluorescent imaging of FE1 cells after 24 Hrs of exposure to 6 µg/mL CuO, NiO, Al_2O_3, or TiO_2 NPs. (A: 100X Fluorescence) Red: α-Tubulin; Green: Lysosomes (Lamp1); Blue: Nucleus (Hoechst 33342); White: particles. (B: 100X LAMP + Darkfield Colocalization) Red: intense darkfield scattering objects; Green: LAMP1; White: Areas of overlap between darkfield and LAMP channels. (C: 100X HSI + SAM overlay): Yellow: Areas with spectral match to the MONP of interest. (D: HSI Hit and Cell Background Spectra) Red: representative spectrum identified as a match through SAM mapping. Black: representative spectrum of cellular background. White arrows identify a co-localizing particle. Yellow scale bars = 10 µm in each image.

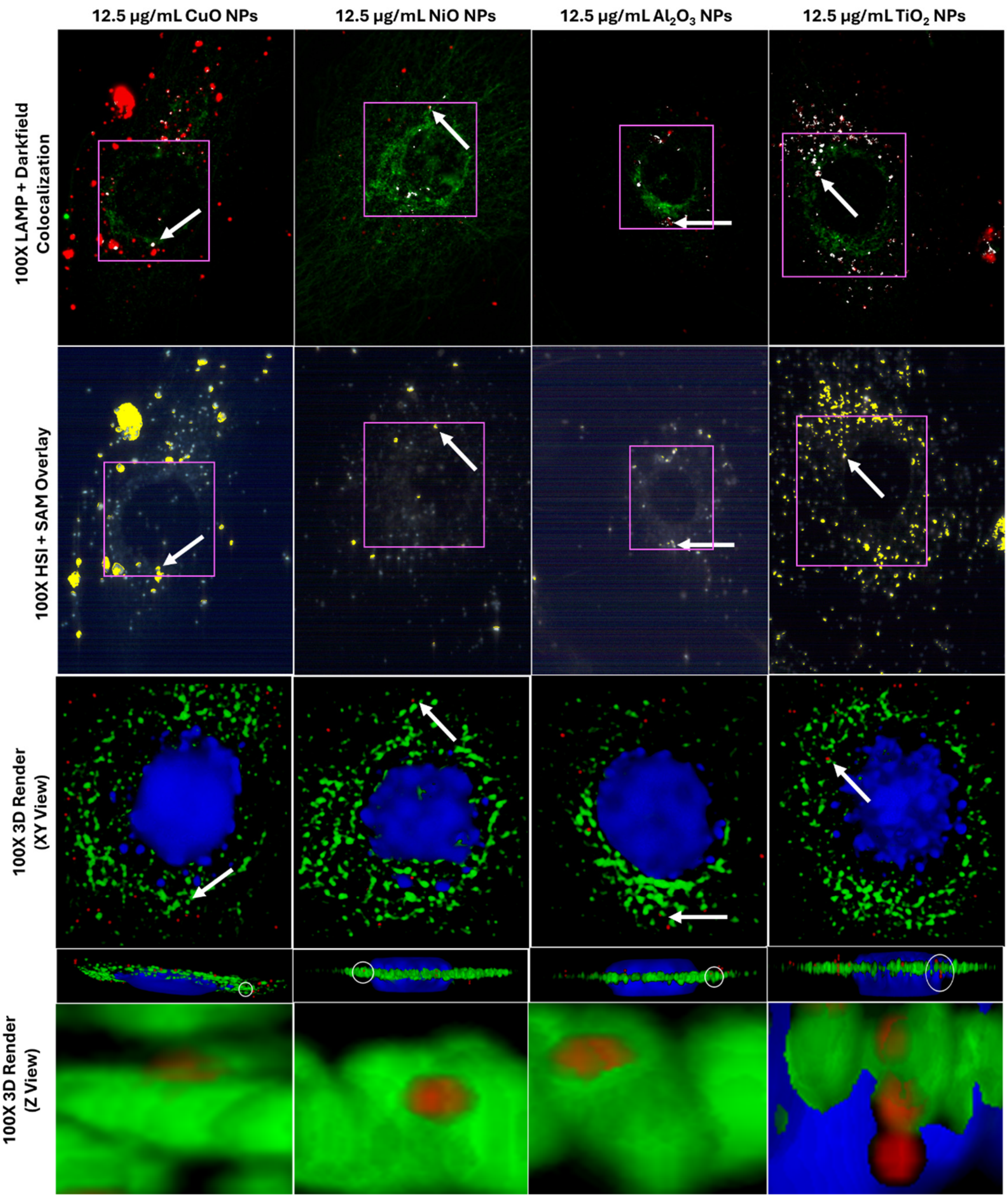

Figure S3. 100X Co-localization of MONPs with LAMP1 signal. Pink boxes indicate areas which were deconvolved for 3D rendering. White arrow identify particles which show co-localization with LAMP1 signal and positive SAM identification as the material of interest. The white circles in the Z-view 3D images identifies the same particle highlighted in 2D, showing that it is in-plane with the Lamp signal and has overlap with the signal in 3 dimensions. (100X LAMP1 + Darkfield colocalization) Green: LAMP1 signal; Red: particle signal; white: areas of signal overlap determined through auto-thresholding. (100X HSI + SAM Overlay) Yellow: SAM identification of MONP. (100x 3D render (XY View)) Green: LAMP1 signal; Red: particles; Blue: Nucleus. (100X 3D Render (Z View)) Inset is an enlargement of the particle highlighted with a white circle.

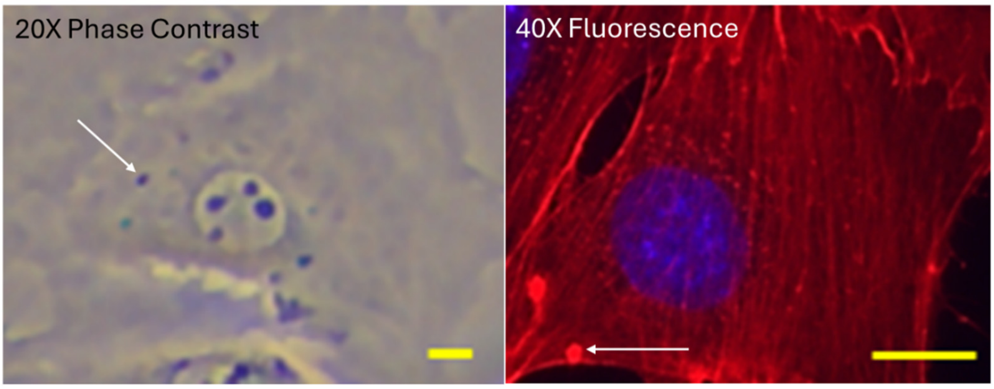

Figure S4. Comparison between cytoplasmic inclusions visible in phase contrast imaging of FE1 cells, and bright actin aggregates found in media control FE1 cells. White arrows: cytoplasmic inclusions and actin aggregates. Scale bars: 10 μm.

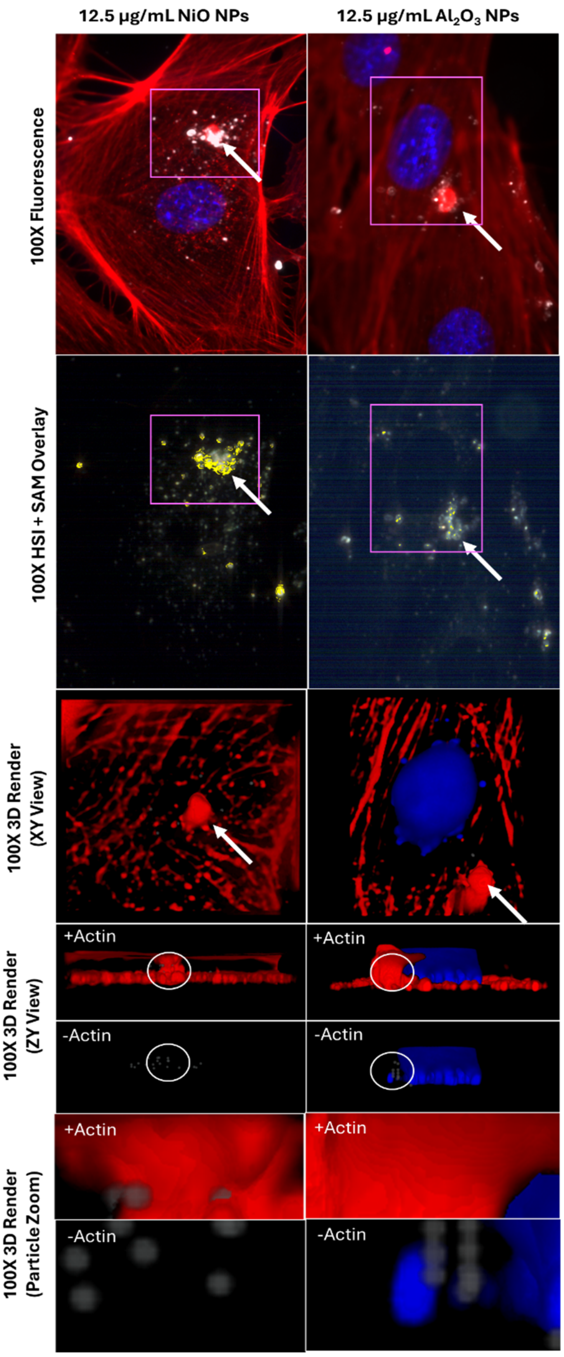

Figure S5. 100X Co-localization of MONPs with actin aggregates. Pink boxes indicate areas which were deconvolved for 3D rendering. White arrow identifies particles which show co-localization with an actin aggregate and positive SAM identification as the material of interest. The white circles in the Z-view 3D images identifies the same particles highlighted in 2D, showing that they are embedded within the aggregate. (100X Fluorescence) Red: F-actin; blue: nucleus; white: strong scattering darkfield objects. (100X HSI + SAM Overlay) Yellow: SAM identification of MONP. (100x 3D render (XY View)) Red: actin signal; White: particles; Blue: Nucleus. (100X 3D Render (Z View)) Inset is an enlargement of the particles highlighted with a white circle.

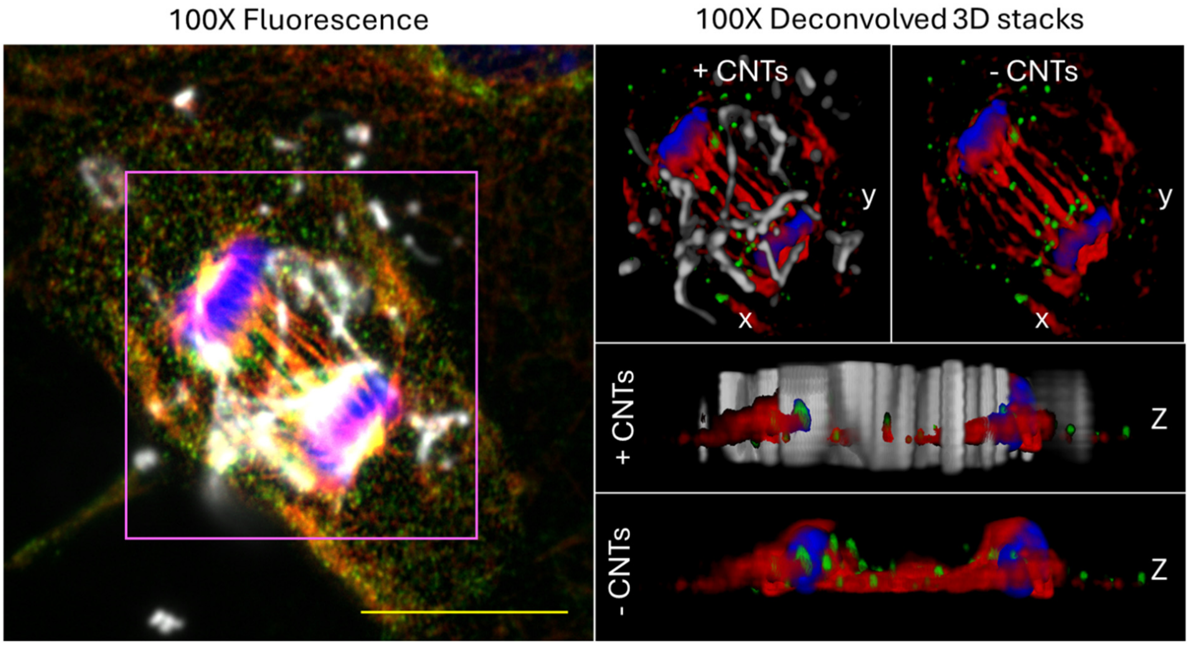

Figure S6. FE1 cell undergoing mitosis following treatment with 100 µg/mL Mitsui-7 for 24 Hrs. (100X Fluorescence) green: Lamp1. Red: α-tubulin. Blue: nucleus. White: Mitsui-7. Purple box: area deconvolved for 3D visualization. Yellow scale bar: 10 µm.
